# Lassa fever–induced sensorineural hearing loss: A neglected public health and social burden

**DOI:** 10.1371/journal.pntd.0006187

**Published:** 2018-02-22

**Authors:** Elizabeth J. Mateer, Cheng Huang, Nathan Y. Shehu, Slobodan Paessler

**Affiliations:** 1 Department of Pathology and Institute for Human Infections and Immunity, University of Texas Medical Branch, Galveston, Texas, United States of America; 2 Department of Medicine, Infectious Disease Unit, Jos University Teaching Hospital, Jos, Plateau State, Nigeria; Oxford University Clinical Research Unit, VIET NAM

## Abstract

Although an association between Lassa fever (LF) and sudden-onset sensorineural hearing loss (SNHL) was confirmed clinically in 1990, the prevalence of LF-induced SNHL in endemic countries is still underestimated. LF, a viral hemorrhagic fever disease caused by Lassa virus (LASV), is endemic in West Africa, causing an estimated 500,000 cases and 5,000 deaths per year. Sudden-onset SNHL, one complication of LF, occurs in approximately one-third of survivors and constitutes a neglected public health and social burden. In the endemic countries, where access to hearing aids is limited, SNHL results in a decline of the quality of life for those affected. In addition, hearing loss costs Nigeria approximately 43 million dollars per year. The epidemiology of LF-induced SNHL has not been characterized well. The complication of LF induced by SNHL is also an important consideration for vaccine development and treatments. However, research into the mechanism has been hindered by the lack of autopsy samples and relevant small animal models. Recently, the first animal model that mimics the symptoms of SNHL associated with LF was developed. Preliminary data from the new animal model as well as the clinical case studies support the mechanism of immune-mediated injury that causes SNHL in LF patients. This article summarizes clinical findings of hearing loss in LF patients highlighting the association between LASV infection and SNHL as well as the potential mechanism(s) for LF-induced SNHL. Further research is necessary to identify the mechanism and the epidemiology of LF-induced SNHL.

## Introduction

Lassa virus (LASV), the causative agent of Lassa fever (LF), is endemic in West Africa, resulting in an estimated 500,000 cases and 5,000 deaths per year [1]. LASV is a member of the *Arenaviridae* family characterized by the appearance of “sandy” ribosomes encapsulated in the virion, as seen in a transmission electron microscope image [2]. The mammalian arenaviruses are divided into Old World and New World based upon geographical, serological, and phylogenetic differences. LASV, along with the prototypical lymphocytic choriomeningitis virus (LCMV) and Lujo virus, are Old World arenaviruses implicated in human diseases [3]. Clinical manifestation of LF ranges from asymptomatic infection to hemorrhagic fever [4]. There are currently no Food and Drug Administration (FDA)-approved vaccinations or treatment for LF. LASV is a Class IA select agent in the United States, thus requiring a high-level containment, biosafety level 4 facility to conduct research [5].

Approximately one-third of LF survivors develop bilateral or unilateral sudden-onset sensorineural hearing loss (SNHL), from which only some patients fully recover [6]. The World Health Organization (WHO) estimates that 368 million people suffer from hearing impairment, with a majority of individuals living in the developing world [7]. Sudden-onset SNHL is defined as damage to the cochlear hair cells or inner ear nerve and diagnosed by a hearing loss of 30 dB or greater over the minimum of 3 different auditory frequencies within 72 hours [8]. Treatment consists of hearing aids or, in severe cases, cochlear implants [8]. However, in the endemic countries, treatment for SNHL is not usually administered [7, 9]. While multiple causes are known for hearing loss in adults, such as ototoxic drugs, occupational hazards, cancer, ear infections, and viral infections (measles, mumps, rubella, and human immunodeficiency virus [HIV]), the incidence of LASV-induced SNHL is largely underestimated in endemic countries [10].

Currently, 37.7 million people are at risk of contracting LASV [11]. With the increase of travel, outbreak risk, and potential use as a biological weapon, it is important to understand the mechanism behind LF-induced SNHL as well as acknowledge the prevalence of SNHL caused by LASV in endemic areas. Understanding the mechanisms will help with the creation of an effective and safe vaccine and therapeutics against LASV as well as treatment for patients with SNHL associated with LF. This review focuses on the current knowledge and highlights the areas of research needed.

### Methodology

A literature review was conducted using electronic databases, including Pubmed/Medline, Google Scholar, and WHO (http://www.who.int). We searched for research articles with the keywords “Lassa fever hearing loss,” “Lassa fever,” “Lassa virus,” “arenavirus sequela,” “hearing loss epidemiology,” “virus hearing loss,” and “sensorineural hearing loss virus.” We analyzed all articles published through June 2017 and included those relevant to the scope of this review.

### Epidemiology, clinical disease, and socioeconomic implications

The first reported case of LF occurred in Lassa, Nigeria, in 1969, when 2 missionaries died from an unknown hemorrhagic fever virus. The virus was later isolated and identified as an arenavirus [12]. LASV is spread through the aerosolization or direct contact of *Mastomys natalensis* urine or feces or through the consumption of food contaminated with its urine or feces [13]. In some villages, hunting *M*. *natalensis* as pests or for consumption of meat is a common practice, resulting in an increased risk of contracting LF [14]. Person-to-person transmission has been reported but typically occurs in nosocomial settings [5].

Traditionally, Nigeria, Liberia, Sierra Leone, and Guinea are recognized as endemic regions, but WHO now considers Benin, Ghana, and Mali endemic regions as well [15]. Serological analysis estimates that up to 50.2%, 55%, and 21% of the population exhibit antibodies to LASV in the endemic countries of Sierra Leone, Guinea, and Nigeria, respectively [16]. The expanding endemic region is due to the ecological similarities and prevalence of *M*. *natalensis* throughout sub-Saharan Africa. Isolated cases have also been reported in the Congo and the Central African Republic, while immunoglobulin G (IgG) antibodies to LASV have been detected in the Ivory Coast [17, 18]. The growing endemic region attests to the outbreak potential of LASV.

A diagnosis of LF can be detrimental to a family in endemic countries. Many patients do not seek appropriate medical care due to the lack of financial resources and distrust of treatments provided by the hospitals [16]. Fetal mortality rate is about 80% if a pregnant mother contracts LF in her third trimester [19]. Miscarriages in endemic countries are attributed to witchcraft, leading to social isolation [16]. The disability of deafness is associated with a stigma of dumbness and results in the inability of a person to succeed economically and socially [9]. Prolonged SNHL can have a dramatic effect on a person’s economic and social status, thus affecting the quality of life. In the developing world, the quality of life is further exacerbated due to the lack of treatment. SNHL individuals are more likely to be unemployed and more often terminated due to tinnitus, vertigo, and the accumulation of sick days. Socially, the lack of understanding and perception of speech lead to social isolation and a decline in communication. With the sudden lifestyle change, many patients suffer from depression, and even those who recover experience debilitating anxiety that the hearing loss will reoccur [7, 9, 20].

WHO estimates the global burden of all hearing loss to be 750 billion dollars. The highest prevalence of hearing loss occurs in sub-Saharan Africa, South Asia, and Asia-Pacific. In Nigeria, 6.7% of the population suffers from hearing loss, costing the country 43 million dollars per year. Only 1% of Nigerians with hearing loss have access to hearing aids [7, 9]. Studies conducted to determine the major causes of hearing loss often neglected the impact of LF, despite its prevalence in the region. An epidemiological study in Benin City, Nigeria, identified the top causes of hearing loss as ototoxicity (15.7%), chronic suppurative otitis media (14.9%), and unknown (10.6%) [21]. In Sierra Leone, a hearing loss risk factor study in children found that 2.7% of hearing-impaired children reported a history of LF by questionnaire, but no LASV antibody testing was performed. No controls reported a history of LF, so an odds ratio could not be calculated [22]. Another hearing loss study in Ilorin, Nigeria, reported that the highest etiological factor of acquired hearing loss in children was febrile illness (18.3%). However, no definite conclusions can be made because febrile illness could be caused by malaria, upper respiratory tract infection, meningitis, typhoid, sinusitis, or LF [23]. To better understand the impact and prevalence of LASV-induced SNHL, patients should be tested for LASV antibodies in future studies.

The actual incidence of annual LF cases remains elusive because a majority of cases, 80%, result in mild symptoms resolving within 1 week [4]. The overall case fatality rate is estimated to be 1%. However, case fatality rates of hospitalized patients rise to 20% and can be as high as 69%, depending on the outbreak [1, 16, 17]. After an incubation period of 1 to 3 weeks, general symptoms of malaise, high fever, and fatigue will develop, followed shortly by pharyngitis, headache, vomiting, retrosternal pain, conjunctivitis, anemia, enlarged lymph nodes, and proteinuria. A majority of cases resolve within 1 to 3 weeks, and only severe cases develop into hemorrhagic fever, occurring approximately 7 days after the onset of symptoms. Severe cases are correlated with high viremia levels and increases in serum levels of liver enzymes, aspartate, and alanine aminotransferases. Symptoms include mucosal and internal bleeding, facial edema, convulsions, and disorientation and ultimately, in many cases, lead to coma and death [4, 16, 24]. During the acute phase of disease, LASV can be diagnosed through reverse transcriptase polymerase chain reaction (rt-PCR) to detect viral ribonucleic acid (RNA). LASV antibodies, typically IgM, develop early in the convalescent stage [25]. Early administration of ribavirin has been shown to decrease mortality by inhibiting LASV replication, but due to its toxic effects, ribavirin is not approved by the FDA for use against LF [26, 27].

### Pathogenesis of LASV-induced SNHL

We retrospectively analyzed 3 hospital-based studies on the clinical course of LF and 2 population studies of LASV-seropositive residents or missionaries performed between 1972 and 1996. Our analysis reveals that an average of 33.2% (4%–75%) of LF survivors developed unilateral or bilateral SNHL [14, 28–31]. These SNHL cases were identified at 10 to 15 days after the onset of LF symptoms or at the convalescent stage of the disease. To confirm the hypothesized association between LF and hearing loss, Cummins et al. performed a case-control study looking at 3 separate groups: hospitalized febrile patients, healthcare personnel and known LASV-seropositive residents, and residents of the Eastern Province of Sierra Leone who experienced sudden onset of deafness [6]. In the first group, 29% of LASV antibody–positive patients developed acute deafness in at least 1 ear at 5 to 12 days after the fever subsided. All patients had seroconversion before the onset of hearing loss. Permanent hearing loss occurred in 17.6% of the LF SNHL patients. None of the LASV antibody–negative febrile patients developed SNHL. In the second group of healthcare personnel and known LASV-seropositive residents, SNHL occurred in 17.6% of those individuals who tested positive for LASV antibody, indicative of previous LASV exposure, compared with only 3% (2 out of 74 ears) of the seronegative controls. In the third group of residents with sudden hearing loss in the Eastern Province of Sierra Leone, 81.2% were serum positive for LASV antibody, compared with 18.8% LASV antibody–positive in the non-SNHL group. In the LASV antibody–positive SNHL group, 71.9% had profound hearing loss in at least 1 ear [6].

This study identified the widespread complication of hearing loss associated with LF even in mild cases. It also highlights that SNHL in endemic countries is more common than originally thought [6]. Based on the known toxicity of ribavirin, an important concern was that SNHL in LF survivors could be an ototoxic effect of ribavirin [27]. However, the prognosis and development of SNHL did not correlate with administration of ribavirin, suggesting that ototoxicity was not the mechanism. The persistence of SNHL despite ribavirin treatment also indicates that reducing viremia levels does not prevent development of SNHL. Overall, viremia levels, liver enzyme levels, or severity of disease did not increase or decrease the risk of development of SNHL. These observations, as well as the convalescent onset of SNHL, led the authors to hypothesize that the pathogenesis of SNHL is not directly caused by the virus infection but may be immune mediated [6].

Since 1972, 6 in-depth case studies in which patients presented with LF and experienced SNHL have been published (Table 1) [28, 30, 32–34]. As was also seen in the Cummins et al. study, only 2 of the cases were treated with ribavirin, whereas the other 4 cases had no specific treatment. The development of SNHL in the absence or presence of ribavirin indicates that the mechanism is not due to otoxocity of ribavirin or viremia levels, thus favoring the immune-mediated mechanism [33]. In case 6, the lack of fever present during hospitalization and increased levels of lymphocytes in the cerebral spinal fluid (CSF) led the authors to hypothesize that the neuropathogenesis of LF could be due to the immune response [34].

**Table 1:** Summary of LF-induced SNHL case studies.

Case No. [Ref.]	Location	Symptoms	Confirmation of LASV	Treatment	Onset of Hearing Loss	Unilateral or Bilateral Hearing Loss	Follow-up of Hearing Loss
1 [28]	Nigeria	Headache, chest pain, diarrhea, vomiting, high fever	LASV detected in serum and serum positive for LASV antibodies	No specific treatment for LF	13 days after onset of fever	Not reported	Present several months later
2 [30]	Liberia	Fever, chills, weakness, diarrhea, cough, lower abdominal and lumbosacral pain, pharyngitis, maculopapular rash on face, headache, lymphadenopathy, periorbital edema, conjunctivitis, vomiting	LASV detected in serum and serum positive for LASV antibodies	No specific treatment for LF	17 days after onset of fever	Unilateral	No follow-up performed
3 [32]	Sierra Leone	Fever, abdominal pain, chills, headache, myalgia, arthralgia, conjunctival injection, lymphadenopathy	Serum positive for LASV antibodies	No specific treatment for LF	Upon entering convalescence phase	Unilateral	Still present upon discharge
4 [33]	Nigeria	High fever, vomiting, abdominal pain, diarrhea, sore throat, difficulty swallowing	rt-PCR	10-day ribavirin	While taking ribavirin	Unilateral	Still present after 4 years; uses hearing aid
5 [33]	Nigeria	High fever	rt-PCR	10-day ribavirin	8 days after onset of fever	Bilateral	Still present after 1 year
6 [34]	Liberia	Fever, nausea, arthralgia, headache, diarrhea, personality changes, encephalitis	LASV detected in serum and CSF	No specific treatment for LF	22 days after onset of symptoms	Bilateral	Still present 2 months later

Abbreviations: CSF, cerebral spinal fluid; LASV, Lassa virus; LF, Lassa fever; rt-PCR, reverse transcriptase polymerase chain reaction; SNHL, sensorineural hearing loss.

Unlike as reported in the Cummins et al. study, cases 4 and 5 experienced SNHL during the acute phase of the disease as opposed to the convalescent phase. The authors of these 2 case studies agreed with the Cummins et al. hypothesis that SNHL is most likely an immune-mediated injury but raised questions due to the prevalence of both unilateral and bilateral hearing loss as well as acute and convalescent onset of SNHL [33]. Bilateral hearing loss is typically associated with an immune-mediated response [35]. The development of SNHL during the acute phase of disease, when the virus is actively replicating, suggests direct viral damage as a mechanism, whereas the convalescent-stage onset of symptoms is likely associated with an immune-mediated injury [10].

After the report of SNHL during the acute phase of the disease, a follow-up case-control study of LF patients with early-onset SNHL in Nigeria was conducted [33, 36]. In this study, 13.5% of patients with LF, confirmed by rt-PCR, developed early-onset bilateral SNHL compared with the 0% for the febrile control group. IgM was detected in 60% of LASV SNHL patients. Interestingly, in this study, it was found that LF patients with SNHL had a higher case fatality rate, 60%, compared with 21.9% for LF patients without SNHL. Due to the lack of all cases exhibiting an antibody response to LASV, as well as a higher case fatality rate, the authors suggested that the mechanism for SNHL was not just immune mediated and proposed direct viral damage as a potential mechanism as well [36]. However, it is important to note that in this study, an early-onset case was defined as development of SNHL within 21 days of acute disease but was not based on seroconversion or entering into the convalescent stage as defined by Cummins et al. [6, 36]. These differences identify the need for common definitions to better identify the onset of SNHL induced by LASV and the need to identify the mechanism and pathology to provide adequate treatment. LF patients who developed SNHL in this study were treated with steroids, hyperbaric oxygen, labyrinthine vasodilators, and vitamin supplements, as well as given hearing aids with no improvements noted [36]. This may suggest that the SNHL induced by LASV causes extensive nerve damage and requires cochlear implants to be corrected [36].

Hypotheses of immune-mediated, direct viral damage, or a combination have been proposed from LF SNHL case and prevalence studies. Understanding the mechanism behind LF-induced SNHL, however, has been hampered due to the lack of autopsy samples or accessibility to biopsy samples from the inner ear. Nevertheless, Yun et al. recently developed an animal model that mimics SNHL associated with LF to alleviate these shortfalls (Table 2) [37]. Mice lacking a functional signal transducer and activator of transcription 1 (Stat1) pathway (Stat1^−/−^) or deficient in alpha/beta gamma interferon receptor (IFN-α/βγ R^−/−^) are used to study LASV pathogenesis [38, 39]. The Stat1^−/−^ mice developed SNHL 16 to 45 days post infection at a rate similar to that of the human disease. Histopathological findings from the inner ears of SNHL Stat1^−/−^ mice showed damage to the spiral ganglion cells and cochlear nerve as well as the thinning of the stria vascularis, distention of the Reissner’s membrane, infiltration of blood cells within the scala tympani, and a minor loss of inner and outer hair cells. Identification of LASV antigen and CD3^+^ lymphocytes was also observed in the damaged regions. Conversely, in the IFN-α/βγ R^−/−^ mice, none developed SNHL although they exhibited higher viral titers compared with the Stat1^−/−^ mice. Both IFN- α/βγ R^−/−^ and Stat1^−/−^ mice stained positive for LASV in the cochlear nerve tissue, but only Stat1^−/−^ mice readily stained for CD3^+^.

**Table 2:** Comparison of LF SNHL in mouse model to LF SNHL in humans.

LF Features	Humans [1, 4, 6, 16, 28–34, 36]	Stat1^−/−^ Mice [37, 38]
Mortality rate	1%	10%
LF symptoms	Fever, fatigue, headache, nausea, anorexia, hemorrhage	Fever, hemorrhage, weight loss
Prevalence of hearing loss	Approximately one-third of survivors	85% of survivors
Onset of hearing loss	Sudden onset Acute phase or convalescent phase	Sudden onset ≥16 days post infection
Severity of hearing loss	Severe to profound	Severe to profound

Abbreviations: LF, Lassa fever; SNHL, sensorineural hearing loss.

One potential mechanism of immune-mediated SNHL is antibody–antigen reaction resulting in the loss of cochlear cells [40, 41]. However, this hypothesis was questioned because some human cases of SNHL develop without the presence of antibodies [36]. The minor damage observed to the cochlear hair cells could indicate direct viral damage to the cochlea. The presence of hearing loss and CD3^+^ cells in Stat1^−/−^ mice versus IFN-α/βγ R^−/−^ mice supports the hypothesis of immune-mediated injury proposed by Cummins et al. [6, 37]. Further research is necessary to clearly identify the mechanism.

LASV primarily targets macrophages, monocytes, and dendritic cells, causing myeloid cell dysregulation. LASV-infected myeloid cells fail to activate the adaptive immune response [24]. In humans, LASV targets the liver, spleen, kidneys, heart, adrenal glands, and bone marrow [25]. LASV has been isolated from the CSF of patients and animal models, demonstrating the ability of LASV to infect the brain [39, 42]. The difference between LF survivors and those who succumb to disease is that survivors often have a strong, early T-cell response [24]. Similar to what has been seen in LCMV infection, an early T-cell response is associated with survival, but overactivation of T cells can lead to immune-mediated damage [5]. A chimeric animal model for LASV infection showed that T cell–mediated immunopathology was contributory to LF pathogenesis. Depletion of CD8^+^ T cells prevented death despite the high viremia levels [43]. The case report of a LF patient’s immune response showed biphasic CD8^+^ T-cell response. The first peak was correlated to virus clearance, whereas the second peak occurred after the virus was cleared at 23 days after the onset of symptoms. Even in the absence of a detectable level of virus, the patient developed lymphadenitis and epididymitis at the time of the second peak of CD8^+^ T cells, aligning with the proposed hypothesis of a T cell–immune-mediated injury leading to SNHL in LASV patients [44]. Future studies are needed to confirm the biphasic pattern of CD8^+^ T-cell response in other patients with LF-induced SNHL. While the neuropathogenesis of LASV remains unknown, studies on animal models and clinical cases have provided evidence for direct viral infection damage or an immune-mediated injury.

An examination of SNHL caused by other viruses can also give insight into the mechanism as well as suggest treatment options for LF-induced SNHL. Measles virus, mumps virus, rubella virus, varicella zoster virus (VZV), cytomegalovirus (CMV), HIV, enterovirus, and hepatitis virus have all been implicated as causative agents of SNHL. However, the rate at which LASV causes SNHL is much higher than reported for any other virus [10, 45]. The proposed mechanism of SNHL for mumps virus, rubella virus, and varicella zoster virus is direct viral damage to the inner ear structures or, in the case of varicella, to the nerves. In measles virus and HIV, the development of SNHL is correlated with severe disease progression, and the proposed mechanism is direct viral damage to the nerves as well as increased susceptibility to bacterial infections. Cases of SNHL due to measles, mumps, rubella, and varicella have drastically reduced since the development and use of the measles/mumps/rubella (MMR)/VZV vaccine [10].

Conversely, the mechanism behind congenital CMV-induced SNHL is believed to be due to the host immune response. Studies with a murine model of CMV infection show that the virus can infect perilymphatic epithelial cells and spiral ganglion neurons but not cochlear hair cells. After viral clearance, a decline in cochlear hair cells was observed, suggesting that CMV-induced SNHL is a result of the immune response. But it remains in question whether the mode of inoculation is representative of human pathogenesis [46]. Another murine CMV model, whose histopathology more closely resembles fetal autopsy samples, showed the loss of the spiral ganglion nerve and an inflamed stria vascularis as marked by the presence of CD3^+^ T cells [47]. However, the most remarkable difference between CMV- and LASV-associated SNHL is the age of onset. Other studies have shown murine CMV-induced SNHL could also be a result of neuronal failure to differentiate or impaired development of the stria vascularis [48]. For CMV, ganciclovir has been shown to prevent progression and even revert SNHL. In all cases, treatment with hearing aids—or in severe cases, cochlear implants—is needed [10].

Sudden-onset SNHL can also be caused by ototoxic drugs whose proposed mechanism is immune-mediated injury. Proinflammatory cytokines interleukin 1β (IL-1β), IL-6, and tumor necrosis factor α (TNF-α) promote the translocation of nuclear factor kappa-light-chain-enhancer of activated B cells (NFκB) to the nucleus and induce the production of reactive oxygen species (ROS). Ultimately, this activates the mitogen-activated protein kinase (MAPK)–c-Jun N-terminal kinase (JNK) pathway, leading to cochlear hair cell apoptosis, as well as blocks the atonal transcription factors Atoh1 and Hath 1, which are responsible for the generation of cochlear hairs [41, 49, 50].

Along with SNHL, other neurological sequelae, including ataxia and acute or chronic neuropsychiatric syndromes, have been associated with LASV but at a much lower rate [51]. Understanding the mechanism behind other neuropathology associated with LF could aid in understanding the mechanism of LF-induced SNHL. In a study to determine the prevalence of neurological deficits due to LF, it was found that 6% of hospitalized patients (survivors or those who succumbed to disease) experienced convalescent-stage ataxia, 6% hearing loss, and 6% neuropsychiatric changes. Cerebellar ataxia occurs after the development of LASV antibodies and can manifest as acute or chronic. The neuropsychiatric disorders reported were insomnia, tinnitus, clinical depression, mania, and asthenia as well as dementia [51]. As was seen in case 6, encephalitis is also a complication of LF [52]. A study to investigate encephalitis caused by LASV found no correlation with the presence of virus or antibodies in the CSF, suggesting a T cell–immune-mediated mechanism. However, LASV has been isolated from encephalitic cases, suggesting that the virus can disseminate to the central nervous system [34, 42, 53]. Another complication for LF survivors is a postinfectious fatigue syndrome. At 3 to 6 months post convalescence, a subset of patients experience debilitating fatigue, which results in loss of productivity [52]. Other complications such as vertigo, not associated with deafness, have been reported as well [30, 54].

While there are no known reports of New World arenaviruses causing SNHL, ataxia occurs in 10% of patients with Argentine hemorrhagic fever—caused by the New World arenavirus Junin—when the patient is treated with immune plasma. This suggests that the ataxia is due to an immune-mediated pathology [55]. The Old World arenavirus LCMV has also been known to cause neurological sequelae, including brain dysfunction and vision impairment, but this is primarily in congenital cases. The mechanism for LCMV-associated neuropathies is believed to be immune mediated specifically due to cytotoxic T-cell response [56].

## Conclusion

Although LF-induced SNHL has been documented, the prevalence and economic impact in endemic regions may be underestimated. Further epidemiological studies are therefore needed to understand the degree of impact as well as address the difference between acute and convalescent SNHL, if it exists. With the socioeconomic impact of SNHL induced by LF, epidemiological studies should also focus on the prevalence in children. While the majority of evidence favors the immune-mediated hypothesis, direct viral damage cannot be ruled out. It would be important to conduct further studies to identify the effect of ribavirin administration on the development of SNHL, the involvement of T cells, and the ability of LASV to replicate in the cochlea to uncover the mechanism. Ultimately, understanding the mechanism of LASV-induced SNHL can provide information for better diagnosis, treatment, and vaccine development as well as insight into other neurological symptoms caused by LASV and other arenavirus family members.

**Key learning points**

- Sudden-onset SNHL occurs in approximately one-third of LF survivors and is not dependent on viremia levels, administration of ribavirin, severity of disease, or liver enzyme levels, suggesting that it could be due to an immune-mediated mechanism. However, further research is required to confirm whether SNHL is caused by direct viral damage, immune-mediated damage, or a combination of both.
- LF-induced SNHL may be an underestimated complication in survivors contributing to the high cost of hearing loss–associated healthcare expenses in endemic countries.
- A STAT1^−/−^ mouse model that replicates SNHL rates seen in humans has been developed. The model showed the infiltration of lymphocytes in the cochlea compared with the IFN-α/βγ R^−/−^ model, which had higher viremia levels but no SNHL or lymphocyte infiltration.

**Top five papers**

1. Cummins D, McCormick JB, Bennett D, Samba JA, Farrar B, Machin SJ, et al. Acute sensorineural deafness in LF. JAMA. 1990;264(16):2093–6. PubMed PMID: 2214077.
2. Yun NE, Ronca S, Tamura A, Koma T, Seregin AV, Dineley KT, et al. Animal Model of Sensorineural Hearing Loss Associated with Lassa Virus Infection. J Virol. 2015;90(6):2920–7. Epub 2015/12/30. doi: 10.1128/JVI.02948-15. PubMed PMID: 26719273; PubMed Central PMCID: PMCPMC4810655.
3. White HA. Lassa fever. A study of 23 hospital cases. Trans R Soc Trop Med Hyg. 1972;66(3):390–401. PubMed PMID: 5046379.
4. Grahn A, Bråve A, Lagging M, Dotevall L, Ekqvist D, Hammarström H, et al. Imported Case of Lassa Fever in Sweden With Encephalopathy and Sensorineural Hearing Deficit. Open Forum Infect Dis. 2016;3(4):ofw198. Epub 2016/09/20. doi: 10.1093/ofid/ofw198. PubMed PMID: 27975074; PubMed Central PMCID: PMCPMC5152670
5. Ibekwe TS, Okokhere PO, Asogun D, Blackie FF, Nwegbu MM, Wahab KW, et al. Early-onset sensorineural hearing loss in Lassa fever. Eur Arch Otorhinolaryngol. 2011;268(2):197–201. Epub 2010/09/01. doi: 10.1007/s00405-010-1370-4. PubMed PMID: 20809263.
